# Arabidopsis bioinformatics resources: The current state, challenges, and priorities for the future

**DOI:** 10.1002/pld3.109

**Published:** 2019-01-04

**Authors:** International Arabidopsis Informatics Consortium, Colleen Doherty, Joanna Friesner, Brian Gregory, Ann Loraine, Molly Megraw, Nicholas Provart, R. Keith Slotkin, Chris Town, Sarah M. Assmann, Michael Axtell, Tanya Berardini, Sixue Chen, Malia Gehan, Eva Huala, Pankaj Jaiswal, Stephen Larson, Song Li, Sean May, Todd Michael, Chris Pires, Chris Topp, Justin Walley, Eve Wurtele

**Keywords:** Arabidopsis, informatics, international collaboration, portal, The Arabidopsis Information Resource

## Abstract

Effective research, education, and outreach efforts by the *Arabidopsis thaliana* community, as well as other scientific communities that depend on Arabidopsis resources, depend vitally on easily available and publicly‐shared resources. These resources include reference genome sequence data and an ever‐increasing number of diverse data sets and data types. TAIR (The Arabidopsis Information Resource) and Araport (originally named the Arabidopsis Information Portal) are community informatics resources that provide tools, data, and applications to the more than 30,000 researchers worldwide that use in their work either Arabidopsis as a primary system of study or data derived from Arabidopsis. Four years after Araport's establishment, the IAIC held another workshop to evaluate the current status of Arabidopsis Informatics and chart a course for future research and development. The workshop focused on several challenges, including the need for reliable and current annotation, community‐defined common standards for data and metadata, and accessible and user‐friendly repositories/tools/methods for data integration and visualization. Solutions envisioned included (a) a centralized annotation authority to coalesce annotation from new groups, establish a consistent naming scheme, distribute this format regularly and frequently, and encourage and enforce its adoption. (b) Standards for data and metadata formats, which are essential, but challenging when comparing across diverse genotypes and in areas with less‐established standards (e.g., phenomics, metabolomics). Community‐established guidelines need to be developed. (c) A searchable, central repository for analysis and visualization tools. Improved versioning and user access would make tools more accessible. Workshop participants proposed a “one‐stop shop” website, an Arabidopsis “Super‐Portal” to link tools, data resources, programmatic standards, and best practice descriptions for each data type. This must have community buy‐in and participation in its establishment and development to encourage adoption.

## 1 INTRODUCTION

*Arabidopsis thaliana* (hereafter Arabidopsis) was the first plant to have its genome completely sequenced. Although many other plants now have fully sequenced genomes, Arabidopsis continues to be the premier reference for plant biology research in a wide range of areas from molecular mechanisms to global ecology. Well over 30,000 researchers world‐wide use Arabidopsis directly or data derived from Arabidopsis to inform their research. Data exchange and data sharing are crucial features for the success of Arabidopsis given its fundamental place in the plant research ecosystem. Continuing development of increasingly sophisticated technologies and the ensuing massive data sets make planning for the future of Arabidopsis bioinformatics of paramount importance.

Following the announcement of the termination of the US National Science Foundation's funding for TAIR (The Arabidopsis Information Resource that has served researchers since 1999), the Arabidopsis community held a series of workshops to discuss future needs with respect to Arabidopsis bioinformatics, resulting in two white papers published in *The Plant Cell* in 2010 and 2012 (International Arabidopsis Informatics Consortium 2010, 2012). The first publication described the need for a new international Arabidopsis bioinformatics initiative: the International Arabidopsis Informatics Consortium (IAIC). The aim of the initiative was to enable plant scientists to develop systems to manage increasing amounts and types of data, and to allow the leveraging of resources, knowledge, and collaborations. Following a strong tradition of international cooperation in the Arabidopsis community, the IAIC envisioned building collaborative teams focused on development of a distributed system of data, tools, and resources. Work resulting from the initiative was intended to be funded by a variety of sources under an international management and scientific advisory board. Thus, the IAIC would need to be dynamic and represent the evolving needs and capacities of the community while reflecting the funding interests of the respective countries.

The second publication resulted from a collaborative “Design Workshop” (International Arabidopsis Informatics Consortium, 2012) that brought biologists and computational experts together to consider community needs to recommend design features for an informatics portal, initially called the “AIP” (Arabidopsis Information Portal), and since renamed “Araport,” to replace and augment TAIR. The overarching goal was that Araport would be the underlying infrastructure of Arabidopsis informatics and would interact with and link to resources across the globe including Arabidopsis data sets generated in individual laboratories, information from other species, and other biological data sets. Additionally, important community‐generated modules would be linked to Araport in a federated approach allowing data, resources and tools generated worldwide to become part of the Arabidopsis Informatics ecosystem. A federated approach was preferred to allow workload, human expertise, innovation, and costs to be shared across many international sites. The idea was that this would produce additional resilience, flexibility, and opportunities to bring together creativity and energy from many places.

**Figure 1:**
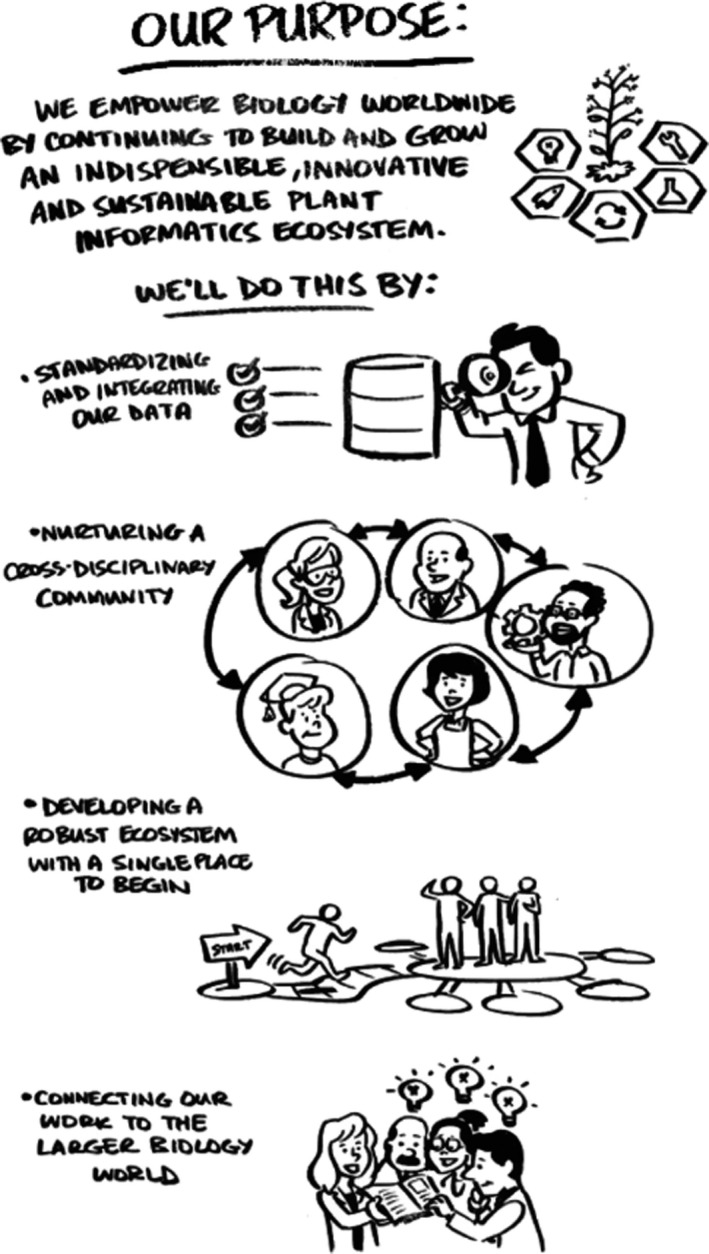
The purpose of the 2018 IAIC workshop. Workshop participants collaboratively defined the overarching purpose for the workshop which focused on improving, extending, and sustaining Arabidopsis informatics efforts, and on establishing & strengthening connections to other research communities. This figure is a representation of the workshop. Image Credit: Filament LLC (theFilament.com)

In the intervening years, Araport.org was established by Chris Town and colleagues, while TAIR continued annotation as a not‐for‐profit organization funded by subscriptions (individual, institutional, and even country‐level, for China and Switzerland). Thus, the two resources now co‐exist and offer complementary functionalities, with Araport focusing on the computational aggregation of diverse resources while TAIR continues to emphasize high quality functional annotation. On the Araport platform, the federated approach was less successful than envisaged, largely due to lack of resources available to individual labs to develop web services to expose and share their data. However, a significant number of such web services do exist, most notably those developed by the Bio‐Analytic Resource (BAR) with funding from Genome Canada along with the native web services that are embedded in ThaleMine, which was adopted as a core component of Araport. Large numbers of new tools and interfaces have been developed in the research community and “omics” data from other plant species are now easy to generate, but this occurs with a risk of fragmentation. Thus, the IAIC decided to hold another workshop to evaluate the current status of Arabidopsis Informatics and chart a course for future research and development (Figure 1).

## 2 THE WORKSHOP

This white paper reports the outcomes from the “2018 Future of Arabidopsis Bioinformatics” workshop funded by an NSF Research Coordination Network (RCN) grant. The workshop's overarching purpose was to reexamine, revise, and extend the original goals for Arabidopsis informatics in light of the advances over the 4 years since Araport was established, and to consider future directions and priorities for Arabidopsis bioinformatics in general. A specific objective was to develop a set of short and mid‐term recommendations for collaborative efforts with respect to bioinformatics tools, resources, infrastructure, and community‐building. First, workshop participants were briefed on results from a community survey of plant bioinformatic needs conducted by the Multinational Arabidopsis Steering Committee (“MASC”) in preparation for the activity (http://arabidopsisresearch.org/images/publications/documents_articles/2018_MASC_BioinfoSurvey.pdf). This was followed by a series of short talks by representatives of the major contributors to the current informatics landscape: Araport, TAIR, BAR, ABRC (Arabidopsis Biological Resource Center) and NASC (the Nottingham Arabidopsis Stock Centre), concluding with a description of the OpenWorm project by Stephen Larson. Next, the workshop's 24 participants were divided into working groups based on interest and experience. Two approaches were utilized in the workshop: the first set of parallel sessions engaged participants in four domain‐specific discussions (Genomes & Comparative Genomics; Genomics: RNA; Proteomics and Metabolomics; Phenomics), while the second set of parallel sessions focused on four cross‐cutting/interdisciplinary bioinformatics topics that emerged, in part, from initial group conversations (Connecting Our Insights & Tools to the Broader Community, Creating & Fostering a Vibrant Community, Standardizing & Integrating Data, and Developing Easy & Robust Tools).

The *cross‐cutting/interdisciplinary* sessions involved a series of discussion questions that varied by group topic; some example questions were as follows:

- What new technologies and approaches have created opportunities to better link data types in the last 5 years? Are there any we should be using in the next 5 years?
- Are there new and/or more robust network analysis tools we might use?
- Is there a plug‐in model for Araport or TAIR that would make it easier to integrate one's own tools?
- What persistent long‐term problems (e.g., data accessibility and sharing, metadata quality, data integration, etc.) did we think would be solved by now, but for which little progress has been made?
- How might we develop data standards?
- How might we adopt standardized pipelines?
- What would it take to standardize metadata description files and files for submission such that all needed information and files are present in standard SRA/GEO data submissions?
- How might new databases and tools enable integration across the different data types discussed?
- How might we encourage different resources to work together to integrate all data types for each gene within a single framework?
- What approaches have we used in the past that haven't worked as well as we hoped?
- Are there new community‐building approaches we might want to try?
- How might we increase community use of Arabidopsis resources for interaction, integration, and data storage?
- How might we encourage and/or incentivize more open access to resources?
- How might we identify additional sources of funding for Arabidopsis portals and what might the community be willing to contribute towards, that doesn't exist now?

### 2.1 Overarching themes that emerged from the workshop

A recurring theme throughout the domain‐specific discussions centered around data: the need for common standards, for robust and easily accessible data repositories, and for methods of data integration and visualization. Standards for metadata are vital. All files (in addition to the raw unprocessed data) used in a publication should be made freely and openly available, and conclusions should be able to be recapitulated by third parties. This is particularly important in emerging areas like phenomics, for which community standards are currently under development.

Some of the challenges the participants identified were related to the expected flood of new and independently annotated genomes; these will require new methods for representation, both in databases (data structures, pan‐genomes) and browsers (multiple/alternate reference frames). They will also require a robust plan for gene ID assignment (including provenance ‐ how does one refer to *AT3G24650* from Col‐0 or Cvi, for example?), both within and across species, to assist in gene tracking and cross referencing.

The availability and deployment of bioinformatic tools were discussed at some length. This may be more complicated than data sharing and accessibility. What are the best repositories for sharing? Tools to better share, integrate and visualize non‐nucleic acid‐based data (e.g., post‐translational modification, metabolomes, etc.) are needed. Can tools be repurposed for different data types without too much effort on the part of developers? The limited success of Araport to generate a large tool repository shows that community buy‐in is still a challenge.

## 3 WORKSHOP RECOMMENDATIONS

### 3.1 Integration of data and tools

Arabidopsis needs a system for standardizing and integrating data that is sustainable and not overly dependent on a few individuals. Thus, it will not be monolithic, but can it be distributed? Standards, ontologies, and formats should be clear, commonly adopted and shared, but there will likely be a need for a committee or oversight entity to lead and monitor this effort. Metadata and provenance will be an important part of this effort, as will the ability to manage experimental replicates. While some standards are fairly well‐established and are consistent across species (e.g., transcriptomics and proteomics), others (e.g., metabolomics) require more development.

There are two objectives to accomplish with respect the integration of data and tools. First is a “PlantHub” (or “Arabidopsis Super‐Portal”) that will serve as a guide and launching pad to find and exploit existing data and tool resources (see Education and Outreach, below). The second is to expand the existing collection of tools while working toward both simplifying their use and making tool integration/concatenation possible (i.e., constructing workflows).

### 3.2 Data formats and standards

There should be a community agreement on ontologies, standards and formats, their usage, and enforcement of these, wherever possible. Certainly, an effort is needed to inform the community of their necessity and value. In all areas (genomics, proteomics, metabolomics, and phenomics) there needs to be robust, well‐structured, and easily accessible data repositories.

There remains a need for a centralized annotation authority to focus on maintaining a “gold standard” (i.e., of the highest quality) in gene and genome annotation. This annotation authority needs to be agile enough to assimilate and disseminate new annotation data sets produced by different groups, e.g., AtRTD2 [Arabidopsis Reference Transcriptome Dataset (Zhang et al., 2017)]. In addition, we need an annotation authority to enforce a rational and consistent naming scheme for genes and gene models. This will truly place Arabidopsis as a leader in the field of genome informatics and foster collaboration across organisms.

We would like to present the Arabidopsis community as a model for genome informatics. However, there are aspects on which we could improve, especially in error correction and versioning. We need a more predictable release schedule for gene annotation and a better versioning approach when new genome assemblies and tools are released. One approach is to borrow ideas and approaches from software development (e.g., semantic versioning with major, minor, and micro releases of gene models and/or genome assemblies). Another area for improvement is to ensure that a rational and consistent naming convention is adopted for annotating new gene isoforms, and that all groups are aware of the convention and will consistently apply it. Also, the development of a robust messaging methodology will be vital for the future of the Arabidopsis informatics community, for example, a combination of BIOSTAR, Twitter (several of the main Arabidopsis informatics sites have Twitter accounts), StackOverflow, and the Arabidopsis listservs, such as Arab‐gen based in the US, and Arab‐UK based in the UK, (as well as possible mechanisms that will be developed in the future).

Data formats and standards are essential for a robust collection of data repositories that can be linked together. Equally important is education and outreach to inform the community on how to manage, expose, and share their data. One priority is the need for new or improved standards in proteomics and metabolomics. These might be achieved by consensus among the major producers/publishers in these fields.

For molecular ecology and population genetics, Arabidopsis is a vitally important model that has the potential to foster gene function annotation for additional plant species. The 1001 Genomes Project catalogs variation in Arabidopsis and provides an extremely robust and dense variant map that can provide a model for other plants. Once again, Arabidopsis research provides a functional dataset for discussion across research communities (e.g., we can now collaborate with the rice genome community on the recent 3000 Rice Genomes Project).

### 3.3 Tool development

There is great need for new ways of browsing genomes that can accommodate multiple entities, whether it be a pan‐genome, a complex of genomes, genomes as a network or even a Google Maps approach. One could navigate between different types of browsing according to the level of detail needed. Concurrently, there is a need for a consensus nomenclature at every level of genome and genome feature.

There should be a clear versioning of software tools. Perhaps this could be exemplified via a paper or “manifesto” outlining a clear and concise means for standards to make data sets and software tools available from all large‐scale genomics analyses. Perhaps we can engage university departments (e.g., electrical engineering and computer science‐type students) in tool development? GitHub appears to have come to the front as the most appropriate repository for tool hosting and sharing, and this should be strongly encouraged.

Arabidopsis, with its well‐annotated genome, exemplifies incredible utility in tool development and implementation. In comparison, in tomato, there is currently no annotation of splice variants, and only one gene model per gene. Arabidopsis should be the model to show other organisms what is possible and suggest ways to do it. A key area of future effort is to move analyses done in Arabidopsis into other species, e.g., as ATTED [a coexpression database (Obayashi, Aoki, Tadaka, Kagaya, & Kinoshita, 2018)], PMR [metabolomics and transcriptomic coexpression database (Hur et al., 2013)] and BAR http://bar.utoronto.ca/ are doing. Outreach to other plant groups to convey vital lessons learned would serve to increase interactions between the various populations and allow leveraging of experience in the Arabidopsis model system for additional important species. For example, the soybean genome exists in two different versions with two different naming conventions, which necessitated a tortuous resolution and identifier conversion process: could those with previous experience in Arabidopsis genomes help to inform the decision process in that community?

To promote sustainability and achieve the objective of more genome version releases and updates, we should pursue greater buy‐in by the Arabidopsis community (perhaps like the OpenWorm model for a “reference” animal) to engage assistance in updating data analysis with the release of new versions of the genome and with tool development (i.e., Cytoscape plugins). It would be helpful to have incentives for community buy‐in (e.g., StackOverflow), which could exist in a new “PlantOverflow” with links from TAIR, Araport and other providers. We recognize that crowdsourcing efforts in bioinformatics have had mixed success. However, there is precedent in the many communities in software and informatics where crowdsourcing and volunteer effort is the primary way that new results are achieved. Open source software is a great example of this and we can learn from these communities’ approaches to develop new sustainability models. For example, we need a way to communicate the “coolness factor” of Arabidopsis work to the larger IT community to spark their interest and engage their creativity and talent in collaboration on research goals. Perhaps a platform for faculty, students, and researchers could be created that would recruit software developers and data science experts and link them to Arabidopsis researchers.

Finally, to ensure broad exposure and utility of community‐developed tools, tools should be integrated into a central plant informatics environment (“PlantHub”, see below). Tool developers would remain responsible for keeping their individual tools updated, but the group responsible for environment maintenance could use the tools to keep analyses that use those tools up‐to‐date for the broader community. This configuration would enable the rapid deployment of tools developed by community members—tools which would be updated by those with the most expertise on their tool‐ and allow the plant bioinformatics environment personnel to focus on running the tools regularly and update various analyses.

Everybody would like to have point and click GUIs (graphical user interfaces) for all informatics resources, both for tools and accessing data. Funding within the “TAIRaport complex” (i.e., a combination of TAIR and Araport) to support development of community tools is desirable. Robust and comprehensive data repositories are a recurring theme. Highly desirable would be a platform that integrates all “omics” data—genomics, transcriptomics, proteomics, phenomics, metabolomics, and other omics.

Going beyond this stage from data integration to predictive models, including use of machine learning/AI and the application of this synthesis with meaningful crop biology, is a long‐term goal. It would be a significant achievement to develop the ability to effectively encourage Silicon Valley (and other) tech platforms to buy‐in or contribute to the (plant) informatics ecosystem.

## 4 WORKSHOP RECOMMENDATIONS FOR EDUCATION AND OUTREACH

As was highlighted in the MASC bioinformatics survey and in‐group discussions at the workshop, two important areas for immediate action are greatly extended education, about both data and tools, and the need to encourage community involvement. Perhaps these can go hand in hand.

There is a real need for a comprehensive “PlantHub/Arabidopsis Super‐Portal” hosted in one or more places (TAIR/Araport/Plantae) with short overviews of all of the resources available and with links out to externally‐hosted resources. This is not “Araport reinvented”. There is no intent to link data, but rather to create a comprehensive guide to all major Arabidopsis resources (for example, an initial set are available at TAIR https://conf.arabidopsis.org/display/COM/Resources). This type of resource would provide a list of APIs, programmatic access to tools and data, descriptions of best practices for metadata/formats/identifiers, and succinctly communicate what is available at each location. This can be organized by resource (seed, clone, etc.) or data type (expression, interaction, etc.); it could be modeled after the Plant Image Analysis website https://www.plant-image-analysis.org, which is curated, has links only, and does not integrate data sets. Each tool would be explained with one or two sentences rather than simply the often‐quirky tool/database name.

The site might include a data formatting wizard, and a Google‐like search (which would return all of the tools that are appropriate for “transcriptomic” data as the search term, for example). Or it could include a single gene exploration page (where dynamic links to participating databases can be generated on the fly, similar to the links available in the “External Link” section on TAIR Gene Locus pages). Another alternative would be an “intelligent agent” search that could return data via web services and weight results according to a researcher's area of interest. Better data integration might be accomplished by more proactive collaborations between major players such as TAIR, BAR, Intact, Araport, and Uniprot, one example being in the area of protein‐protein interactions, as there is no canonical database encompassing all Arabidopsis interactions.

For every aspect of the way forward, a single body that represents Arabidopsis Informatics is needed—perhaps some form of merger between MASC and IAIC, noting that MASC comprises the major global Arabidopsis‐representing bodies (for example, the North American Arabidopsis Steering Committee, NAASC, and GARNet in the UK). As a starting point, one could create a “Slack” channel focused on Arabidopsis informatics with sub‐pages on each of the major topics that are the subject of this meeting/report. The community should be made aware of the existence of this method of communication and encouraged to participate and contribute.

This informatics community needs a continuing/renewable voice; perhaps a distillation of the yearly MASC report published, for example, in *Plant Direct*. Currently, the MASC report does a good job of outlining all of the new Arabidopsis resources produced/updated during the preceding year, but the report tends to be less effective in reaching the broader community due to its lower visibility. The various MASC subcommittees should contribute to a summary publication which could include a table of various tools and data resources. In addition to providing a succinct and effective way to share resources TO the community at large, the publication could include instructions on how to submit resources FROM the community, e.g., a table constructed like so: “If you have [type of] data, this is what you should call it, how you should format it, and this is where you should put it”. Such efforts would engage the broader community in sharing resources and could result in more consistency in how resources and tools are presented to the community. Ideally, these tables and instructions would also provide useful information to developers at the beginning stages and facilitate more consistent approaches in the tool and resource development process.

The proposed “PlantHub/Arabidopsis Super‐Portal” site should provide the typical Arabidopsis researcher with clear instructions such that participants at all career and experience stages can engage. It should provide examples of data and resources and clearly state how researchers can contribute these items. For example, a question a user might have is “How can we get our RNA‐seq data into BAR?”

In summary, when the community has a “one‐stop shop” for quickly finding informatics resources and for contributing to these resources, this will create a positive feedback‐loop of engagement that will enable amplification of tools and resources from a broader set of participants, increase knowledge and tool sharing, and underpin and extend collaborative research, education, and practice.

## APPENDIX 1

### Contributors

Primary Contributors: workshop organizers and significant contributors to the final report; Secondary Contributors: workshop attendees and report section contributors.

**Table:**

Name	Institution	Email	Country	ORCID
Primary contributors				
Colleen Doherty	North Carolina State University	cjdohert@ncsu.edu	USA	0000‐0003‐1126‐5592
Joanna Friesner	UC Davis and NAASC	jdfriesner@ucdavis.edu	USA	0000‐0002‐6799‐7808
Brian Gregory	University of Pennsylvania	bdgregor@sas.upenn.edu	USA	0000‐0001‐7532‐0138
Ann Loraine	UNC Charlotte	Ann.Loraine@uncc.edu	USA	0000‐0002‐8365‐0177
Molly Megraw	Oregon State University	megrawm@science.oregonstate.edu	USA	0000‐0001‐6793‐6151
Blake C. Meyers	Donald Danforth Plant Science Center	BMeyers@danforthcenter.org	USA	0000‐0003‐3436‐6097
Nicholas Provart	University of Toronto	nicholas.provart@utoronto.ca	Canada	0000‐0001‐5551‐7232
R. Keith Slotkin	Donald Danforth Plant Science Center	kslotkin@danforthcenter.org	USA	0000‐0001‐9582‐3533
Chris Town	J. Craig Venter Institute	cdtown@jcvi.org	USA	0000‐0003‐4653‐4262
Secondary contributors				
Sarah M. Assmann	The Pennsylvania State University	sma3@psu.edu	USA	0000‐0003‐4541‐1594
Michael J. Axtell	The Pennsylvania State University	mja18@psu.edu	USA	0000‐0001‐8951‐7361
Tanya Berardini	TAIR and Phoenix Bioinformatics	tberardi@arabidopsis.org	USA	0000‐0002‐3837‐8864
Sixue Chen	University of Florida	schen@ufl.edu	USA	0000‐0002‐6690‐7612
Malia Gehan	Donald Danforth Plant Science Center	mgehan@danforthcenter.org	USA	0000‐0002‐3238‐2627
Eva Huala	TAIR and Phoenix Bioinformatics	huala@phoenixbioinformatics.org	USA	0000‐0003‐4631‐7241
Pankaj Jaiswal	Oregon State University	jaiswalp@science.oregonstate.edu	USA	0000‐0002‐1005‐8383
Stephen Larson	OpenWorm Foundation	stephen.larson@gmail.com	USA	0000‐0001‐5397‐6208
Song Li	Virginia Tech	songli@vt.edu	USA	0000‐0002‐8133‐3944
Sean May	NASC and University of Nottingham	sean.may@nottingham.ac.uk	UK	000‐0001‐5282‐3250
Todd Michael	J. Craig Venter Institute	tmichael@jcvi.org	USA	0000‐0001‐6272‐2875
Chris Pires	University of Missouri	piresjc@missouri.edu	USA	0000‐0001‐9682‐2639
Chris Topp	Donald Danforth Plant Science Center	ctopp@danforthcenter.org	USA	0000‐0001‐9228‐6752
Justin Walley	Iowa State University	jwalley@iastate.edu	USA	0000‐0001‐7553‐2237
Eve Wurtele	Iowa State University	mash@iastate.edu	USA	0000‐0003‐1552‐9495
