# Supplementary material for: Arabidopsis bioinformatics resources: The current state, challenges, and priorities for the future
Source: Plant Direct. 2019 Jan 4;3(1):e00109. doi: 10.1002/pld3.109 (PMC6508773; doi:10.1002/pld3.109)
Supplement: Supplementary file 1 [file PLD3-3-e00109-s001.pdf]

Response to Editors' and Reviewers' comments:

1. The report needs a real abstract that provides a summary of their recommendations. The current abstract is merely part of the introduction.

>>> We have modified the abstract to include more details of the recommendations. In fact, we have quite extensively revised the paper to fix a number of small issues that we had noticed in reading the paper. We think that it is much improved in this new version.

2. Line 53: "Design Worksho[1]p". [1] is at the wrong place.

>>> Fixed, thank you.

3. Line 217: "As an example, perhaps a paper / "manifesto" outlining a clear and concise means of the standards of making the data sets and software tools available from all large-scale genomics analyses." This sentence has no verb.

>>> Fixed, thank you.

4. Providing a list or table of external data sources/apps already integrated into the platform would be good to demonstrate the different data types already covered by Araport. Currently, this information is a bit scattered throughout the paper. The nice developments already made should be highlighted!

>>> Fixed, thank you. We have replaced the link to the Google Doc at the end to a more dynamics page that is hosted on TAIR.
